# Growth Recovery After Fetal Growth Restriction: A 10-Year Follow-Up of Term-Born Children

**DOI:** 10.3390/nu18020243

**Published:** 2026-01-13

**Authors:** Anca Adam-Raileanu, Alin Horatiu Nedelcu, Ancuta Lupu, Viorel Țarcă, Laura Bozomitu, Lorenza Forna, Ileana Ioniuc, Cristina Maria Mihai, Tatiana Chisnoiu, Elena Țarcă, Ionela Daniela Morariu, Emil Anton, Bogdan Puha, Vasile Valeriu Lupu

**Affiliations:** 1 Grigore T. Popa University of Medicine and Pharmacy, 700115 Iasi, Romania; anca.adam-raileanu@umfiasi.ro (A.A.-R.); alin.nedelcu@umfiasi.ro (A.H.N.); laura.bozomitu@umfiasi.ro (L.B.); lorenza.forna@umfiasi.ro (L.F.); ileana.ioniuc@umfiasi.ro (I.I.); tarca.elena@umfiasi.ro (E.Ț.); ionela.morariu@umfiasi.ro (I.D.M.); emil.anton@umfiasi.ro (E.A.); bogdan.puha@umfiasi.ro (B.P.); vasile.lupu@umfiasi.ro (V.V.L.); 2 Faculty of Medicine, Apollonia University, 700511 Iasi, Romania; 3 Faculty of Medicine, Ovidius University, 900470 Constanta, Romania; cristina2603@yahoo.com (C.M.M.); tatiana_ceafcu@yahoo.com (T.C.)

**Keywords:** fetal growth restriction, intrauterine growth restriction, catch-up, recovery, term-born

## Abstract

**Background/Objectives**: Fetal growth restriction (FGR) describes the situation of a fetus that fails to reach its genetic growth potential. Postnatal catch-up growth represents a central adaptive process, yet its timing and magnitude vary widely and may influence one individual’s state of health and later metabolic risk. This study aimed to characterize longitudinal growth trajectories from birth to 10 years in children born at term, affected antenatally by growth restriction, with a particular focus on the influence of sex and FGR severity on catch-up growth. **Methods**: We conducted a retrospective observational study including 170 term-born children with documented FGR, admitted to a tertiary pediatric center between 2019 and 2023. Anthropometric data (weight, length/height, BMI) at birth, 1, 2, 5, and 10 years were converted to World Health Organization (WHO) age- and sex-adjusted z-scores. Catch-up growth was defined as an increase of >0.67 SD. Participants were stratified by sex and FGR severity (moderate: 10th–3rd percentile; severe: <3rd percentile). **Results**: Severe FGR infants exhibited significantly lower birth anthropometrics but demonstrated more pronounced early catch-up in weight and length at 1 and 2 years (*p* < 0.01). By 5 and 10 years, growth trajectories converged between severity groups, with no differences in BMI at any age. Sex influenced absolute anthropometric values but not the probability of achieving catch-up growth. **Conclusions**: Among term-born FGR infants, severity—but not sex—shapes early postnatal growth. Despite early deficits, most children achieved substantial catch-up, underscoring the need for careful monitoring to support healthy, proportionate growth and mitigate subsequent metabolic risk.

## 1. Introduction

Fetal growth restriction (FGR), a pathology that is also known as intrauterine growth restriction (IUGR) or fetal growth retardation, refers to a detrimental limitation of the fetus’s ability to achieve its genetic growth potential [1,2]. Despite current advances in modern medical technology, it remains an important cause of perinatal morbidity and mortality worldwide [3,4]. According to available epidemiological data, approximately 5–10% of pregnancies are affected, though its prevalence is estimated to be higher in low- and middle-income countries where risk factors such as maternal undernutrition, infections, and anemia are still common [5,6]. Regarding its underlying physiopathological features, FGR results from a complex interplay of maternal, placental, and fetal factors that compromise both oxygen and nutrient transfer to the developing fetus [7,8,9,10]. Among all these elements, placental dysfunction represents the central mechanism of FGR. More precise structural and molecular alterations within the placenta impair maternal–fetal exchange processes, leading to chronic fetal hypoxemia and deprivation of nutrients [11,12].

The long-term significance of these early life disturbances extends beyond birth. In fact, there is an impressive body of literature that offers proof of how intrauterine growth patterns influence lifelong health outcomes [13,14,15,16]. The “Developmental Origins of Health and Disease” (DOHaD) hypothesis was first advanced by the British epidemiologist David Barker in 1986 [17]. It highlighted the idea that the association of an adverse intrauterine environment and a restricted fetal growth might link individuals to an increased risk of cardiovascular and metabolic events later in life [18,19,20,21]. Over the last decades, this concept has become a cornerstone for our understanding of early life influences on adult state of health [17,18].

FGR represents a subject of general interest, as it affects health far beyond the neonatal period. A growing body of evidence links restricted fetal growth to long-term neurodevelopmental, metabolic, and cardiovascular disease [22,23,24,25,26]. FGR also sets the stage for long-term cardiometabolic vulnerability. Prenatal undernutrition can permanently alter vascular structure, insulin sensitivity, and adipose regulation [27,28,29,30]. In one of their many studies, Barker and colleagues [18] observed that individuals that were affected by a slow fetal growth but experienced a rapid postnatal catch-up period were more likely to develop coronary disease in adulthood. Morrison et al. [27] and Crispi et al. [24] further demonstrated that fetal adaptive responses to placental insufficiency—such as preferential blood flow redistribution to vital organs and hormonal adjustments—may predispose individuals to later development of insulin resistance, visceral adiposity, and arterial hypertension. Emerging evidence from experimental and clinical investigations indicates that postnatal catch-up growth following FGR perturbs pancreatic islet architecture [31] and induces sustained inflammatory responses and metabolic dysregulation within adipose tissue [28,32,33].

FGR hallmarks can also be detected throughout adolescence. Flores-Guillén et al. [34] observed that FGR adolescents in indigenous Mexican populations exhibited higher rates of both stunting and overweight, illustrating how early patterns of growth interact with later nutritional exposure. Such findings underscore the multifactorial nature of postnatal outcomes, shaped by genetic, socioeconomic and environmental factors. The fetal origins hypothesis has also expanded its concept to include emerging associations between FGR and non-alcoholic fatty liver disease (NAFLD) [35], metabolic syndrome [36,37,38], and other chronic medical entities, reiterating the condition’s long-term clinical relevance [39].

Catch-up growth represents a key adaptive phenomenon that occurs as a postnatal response to FGR. Clinically it appears as an accelerated growth phase following a period of intrauterine constraint [40,41]. It seems that by two years of age, approximately 85% of small-for-gestational-age (SGA) infants exhibit partial or complete compensatory growth [41,42]. However, both the magnitude and timing of this compensatory process have crucial implications for subsequent adult state of health. If early infancy catch-up has been associated with insulin resistance, excessive fat deposition and elevated cardiometabolic risk in subsequent life course [43,44,45], an incomplete catch-up can favor a short stature persistence or can open the path to an altered body composition [46,47]. As in a “grow now, pay later” paradigm [48], immediate survival comes with important future metabolic costs. Human and animal model research reveals that early restoration of fat mass without the existence of a proportional gain of lean mass could be considered as a characteristic feature in FGR weight recovery [49,50,51]. Furthermore, experimental models suggest that accelerated growth can promote ectopic lipid accumulation and adipocyte hypertrophy [32,50].

Tracking growth trajectories from birth through childhood is therefore essential to our comprehension of FGR recovery and how it relates to future health outcomes. Different elements including nutritional, endocrine and genetic factors exert a substantial influence on FGR-affected children’s weight and height growth patterns, as reported by several longitudinal cohort studies [29,32,52,53]. Given the potential for substantial long-term morbidity, the early identification of aberrant growth patterns suggestive of metabolic dysregulation or persistent growth failure is imperative for healthcare professionals, in their attempt to prevent or at least slow down the progression towards later cardiometabolic disease. The use of World Health Organization (WHO) child growth charts [54] enables continuous monitoring, offering an efficient and accessible surveillance tool during childhood. Integrating prenatal and postnatal data offers opportunities to develop predictive models and personalized strategies for early life growth disorders, with meaningful implications for ongoing patient management strategies.

The present study offers several innovative contributions to the understanding of postnatal growth in children affected by FGR. First, it focuses exclusively on term-born children with antenatally documented FGR, thereby minimizing the confounding influence of prematurity. Although its effects frequently overlap with those of FGR, prematurity is acknowledged as an independent determinant of postnatal growth and body composition disturbances [31,55]. Distinguishing the precise role of FGR in childhood or adult outcomes can be methodologically difficult. Preterm infants are subject to additional neonatal morbidities, nutritional difficulties, and various environmental stressors that independently affect growth [56,57]. To separate the effects of FGR from the confounding effects of prematurity, the current study was focused only on children born at term with a documented history of intrauterine growth retardation.

Second, the study provides a longitudinal evaluation of growth trajectories from birth to 10 years of age, encompassing critical developmental periods that are often insufficiently explored in prior research. By including multiple standardized assessment points, the analysis captures both early catch-up growth and longer-term growth convergence.

Third, growth was assessed using WHO age- and sex-adjusted z-scores, and catch-up growth was defined using a clinically meaningful threshold (>0.67 SD), facilitating both statistical robustness and clinical interpretability. This approach ensures comparability across ages and alignment with routine pediatric growth monitoring practices [54].

Additionally, classifying FGR according to severity represents a critical yet underexplored dimension in the evaluation of postnatal catch-up growth. Although FGR is widely recognized as a heterogeneous condition, many longitudinal studies assess growth outcomes by grouping all growth-restricted infants together, without accounting for the degree of intrauterine growth impairment [34,46]. This approach may obscure important biological and clinical differences in postnatal growth trajectories.

Severity-based classification reflects the extent and chronicity of intrauterine nutritional and oxygen deprivation, which are central determinants of fetal adaptive responses [2]. Severe FGR is more often associated with prolonged placental insufficiency, altered endocrine signaling, and permanent changes in body composition and metabolic programming. In contrast, moderate FGR may represent a milder or later-onset form of growth restriction, with greater potential for postnatal recovery [6,26]. Without stratification by severity, these distinct intrauterine phenotypes cannot be adequately differentiated.

Furthermore, emerging evidence suggests that the magnitude and timing of catch-up growth may be inversely related to the degree of fetal growth restriction, with severely growth-restricted infants demonstrating more pronounced early catch-up but also potentially higher long-term cardiometabolic risk. However, data supporting this hypothesis remain limited, particularly in term-born populations, as most studies include mixed gestational ages or focus on early infancy alone [16,22].

By incorporating a severity-based classification using standardized birth weight percentiles (<3rd percentile for severe FGR; 3rd–10th percentile for moderate FGR), the present study enables a more granular analysis of growth dynamics across childhood [2,34]. This approach improves the interpretability of longitudinal growth patterns and allows for the identification of critical periods during which compensatory growth is most likely to occur.

Importantly, severity stratification has direct clinical implications. Infants with severe FGR may require closer anthropometric surveillance and tailored nutritional guidance, whereas those with moderate FGR may follow a different growth and risk trajectory [35,44]. Understanding these distinctions supports more individualized follow-up strategies and may help clinicians balance the benefits of catch-up growth against the potential risks of excessive or disproportionate weight gain.

Altogether, current evidence indicates that FGR is not merely a perinatal event but a condition shaping health throughout life. By analyzing postnatal growth trajectories in children with a history of FGR, the present study seeks to advance current knowledge and evaluate their implications for maintaining healthy growth and preventing future metabolic disturbances.

## 2. Materials and Methods

### 2.1. Study Design

This study was designed as a retrospective observational analysis. Its main objective was to evaluate postnatal growth trajectories of children born at term, antenatally diagnosed with FGR. Data were obtained from archived medical records of patients admitted for continuous hospitalization to Saint Mary Children Hospital in Iași, Romania, over a five-year period, from 1 January 2019 to 31 December 2023. The hospital serves as a tertiary referral facility for the northeastern part of the country, providing complex pediatric and neonatal care. Institutional approval for ethical conduct in research was obtained from the Ethics Committee of the Saint Mary Children Hospital, Iași (31602/26 October 2023) and by the Ethics Committee of the Grigore T. Popa University of Medicine and Pharmacy, Iași, Romania (383/27 January 2024).

### 2.2. Participants

**Figure 1:**
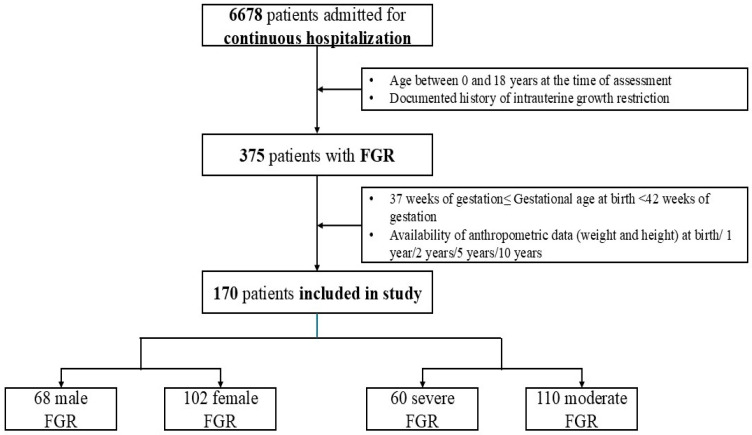
Schematic study group selection. FGR—Fetal growth restriction. Continuous hospitalisation: the patient remained in hospital under medical supervision for at least 24 h.

Inclusion criteria included: age between 0 and 18 years at the time of assessment, a documented history of intrauterine growth restriction, gestational age at delivery between 37 weeks and 42 weeks of gestation, availability of anthropometric data (weight and height) at birth/1 year/2 years/5 years/10 years, as presented in Figure 1.

Information was extracted from hospital observation charts and patient electronic records. For each subject, the following variables were collected and analyzed: demographic data (age, sex, living environment—urban/rural), perinatal characteristics (birth weight, birth length, gestational age, and type of delivery), feeding history in the first six months of life (exclusive breastfeeding, formula feeding, or mixed feeding), anthropometric data (weight and height at birth, 1 year, 2 years, 5 years, and 10 years), and maternal factors (maternal age at delivery and level of education).

The Body Mass Index (BMI) was calculated using the standard formula: BMI = Weight (kg)/([Height (m)]^2^). Thereafter, z-scores, adjusted for age and sex, were determined for weights, lengths, and BMI at birth and at ages 1, 2, 5, and 10 years for all children, in accordance with the WHO standard charts for growth. Catch-up was considered significant when there was an increase of more than 0.67 in weight, length, or BMI z-scores during any of the assessed intervals. The threshold of 0.67 was selected because it corresponds to the width of every percentile segment described on most standardized pediatric growth charts, thereby providing a clinically meaningful indicator of upward crossing between percentile bands. According to the WHO interpretation of BMI for age (5 to 19 years) chart, obesity was defined as an BMI value of >+2SD.

In order to facilitate a complex analysis, we used two classifications of the subjects included in research. First, participants were categorized by sex (male vs. female). Second, they were classified based on the severity of FGR: moderate FGR, defined as birth weight between the 10th and 3rd percentiles according to the WHO growth standards, and severe FGR, defined as birth weight below the 3rd percentile.

### 2.3. Statistical Analysis

The statistical analysis was performed using IBM SPSS Statistics software (version 26.0). Microsoft Excel 2019 was used for data entry and for obtaining descriptive charts. Normality of continuous variables was assessed with the Shapiro–Wilk test and summarized as the mean ± SD; categorical variables were presented as frequencies. Group comparisons between sexes and between moderate and severe FGR were performed using independent *t* tests for continuous variables and Chi-square or Fisher’s exact tests for categorical variables. Longitudinal changes in anthropometric Z-scores were examined using repeated-measures ANOVA, with sex or FGR severity as between-subject factors. Greenhouse–Geisser corrections were applied when sphericity was violated. Catch-up growth was defined as an increase >0.67 in Z-score, and differences in catch-up proportions were assessed using Chi-square tests. Logistic regression was used to evaluate predictors of elevated Z-scores and catch-up growth, with odds ratios (OR) and 95% confidence intervals reported. Statistical significance was set at *p* < 0.05.

## 3. Results

### 3.1. Sample Characteristics

According to our protocol, 170 children fulfilled all the required criteria and were included in the study (Figure 1). As described in Table 1, 40% of them were males and 60% females. When stratified by growth status, 64.6% were classified as having moderate FGR, while 35.3% had severe FGR.

Overall, birth weight and length of moderate and severe FGR resulted in significant differences between the two categories, while gestational age, maternal age, sex distribution, and feeding patterns were similar across groups. The very small *p*-values for birth weight and birth length indicate large effect sizes and highly significant differences between moderate and severe FGR birth anthropometrics.

### 3.2. Sex-Based Comparison Results (Male vs. Female)

**Figure 2:**
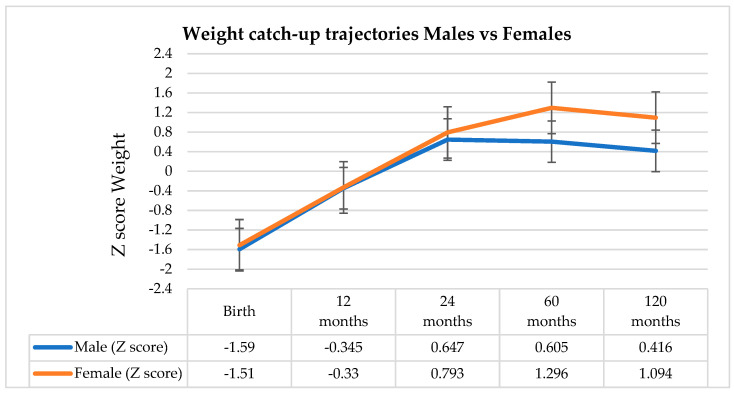
Comparison between mean weight z-scores at ages 1 year, 2 years, 5 years and 10 years, according to patients’ gender.

At birth, mean weight, length, and BMI did not differ significantly between males and females (*p* > 0.05 for all comparisons). However, differences emerged during postnatal development. Male infants exhibited significantly higher mean body weight at 1 year (*p* < 0.001) and 2 years (*p* = 0.02), although this pattern reversed by 10 years, when females demonstrated higher weight compared with males (*p* = 0.03) (Table 2). Mixed ANOVA with repeated measures showed a significant main effect of time on weight Z scores, F(1.932; 324.554) = 48.96, *p* < 0.001; η^2^ = 0.23. The interaction between time and sex was also significant, F(1.932; 324.554) = 4.50, *p* = 0.013; η^2^ = 0.03, indicating different developments over time between boys and girls. The significant interaction between sex and time (*p* = 0.013) reveals fundamentally different recovery trajectories. With similar growth patterns of weight until the age of 2, girls experience an explosive acceleration between ages 2–5, significantly outperforming boys and maintaining this superiority up to age 10 (Figure 2).

In a similar manner, males presented higher BMI values at 1 year (*p* < 0.001) and 2 years (*p* = 0.01), whereas females had a higher BMI at 10 years (*p* = 0.002) (Table 2). Regarding linear growth, males were significantly taller than females at 1 year (*p* = 0.003), but no statistically significant differences were found at other ages (Table 2).

**Figure 3:**
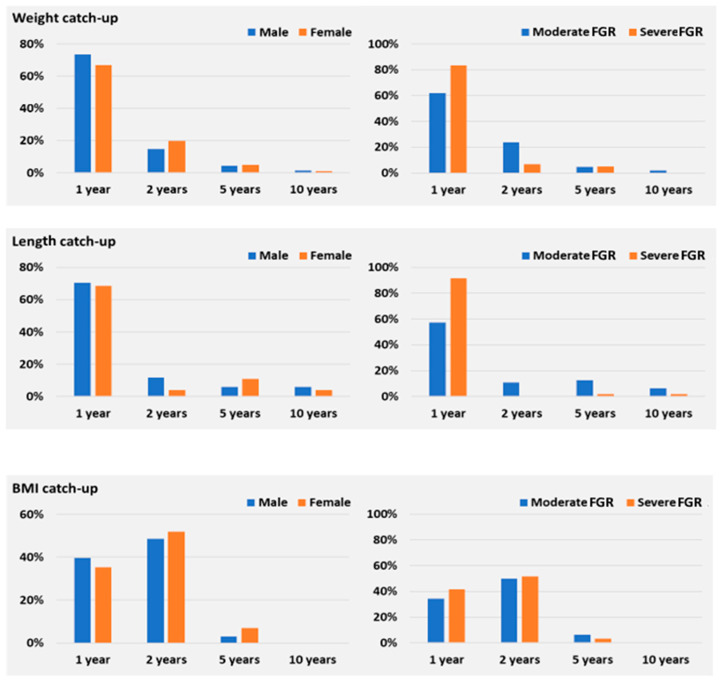
Catch-up for non-cumulative weight, length, and BMI (male vs. female or moderate vs. severe FGR), with repeated measures assessment at 1, 2, 5 and 10 years.

**Figure 4:**
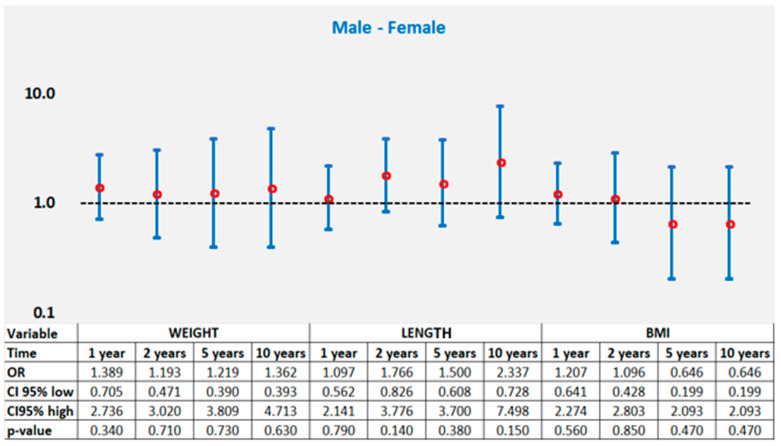
Results of the logistic regression model for catch-up for cumulative weight, length, and BMI for time intervals of 1, 2, 5 and 10 years (univariate analysis).

No significant sex differences were observed in the proportion of children achieving catch-up growth in weight, length, or BMI during their first 10 years of life. Despite males achieving a modest advance in mean weight at 1 year of age (*p* < 0.001), weight catch-up occurred in 73.5% of males and 66.7% of females (*p* = 0.34), and both sexes displayed similar patterns at 2, 5, and 10 years (all *p* > 0.05) (Table 2, Figure 3 and Figure 4).

Catch-up in BMI showed no sex-specific differences, with recovery converging by age 2 and remaining stable thereafter through 10 years (91.2% vs. 94.1%, *p* = 0.47) (Table 3, Figure 4). Length recovery did not differ between boys and girls at any assessment, with catch-up achieved in 70.6% vs. 68.6% at 1 year (*p* = 0.79), rising to 94.1% vs. 87.3% at 10 years (*p* = 0.15). Although males showed slightly higher percentages at each interval, none reached statistical significance (Table 4, Figure 3). Overall, sex was not associated with differences in catch-up growth across any domain or time point.

We observed in most cases that, regardless of the observation time (12, 24, 60 or 120 months), increased Z values (above 2) predominated in female patients compared to male patients for all analyzed indicators (weight, height and BMI). The difference between the two sexes in terms of Z scores for weight was statistically significant for the weight of patients observed at 60 months (χ^2^ = 8.722 with *p* = 0.003). In conclusion, female sex is a significant and strong risk factor for recording increased values of Z scores for weight in patients with FGR at the age of 60 months (OR = 3278; 95% CI: 1453–7392).

Also, at the age of 60 months, we recorded an almost double percentage of obese female patients who had BMI Z-score values above 2 compared to male patients, the difference being in this case only a marginal significance (χ^2^ = 3.748 with *p* = 0.053) (Table 4).

### 3.3. Severity-Based Comparison (Moderate vs. Severe FGR)

FGR severity was highly related to growth outcomes, particularly throughout early life. As expected, children with severe FGR had substantially lower birth weight (2.32 ± 0.16 kg vs. 2.61 ± 0.10 kg; *p* < 0.001) and shorter birth length (43.57 ± 1.13 cm vs. 46.18 ± 1.14 cm; *p* < 0.001) compared with those with moderate FGR (Table 2).

Postnatally, however, differences between moderate and severe FGR groups diminished and no significant differences in mean weight, length, or BMI values were observed at 1, 2, 5, or 10 years (all *p* > 0.05) (Table 2).

**Figure 5:**
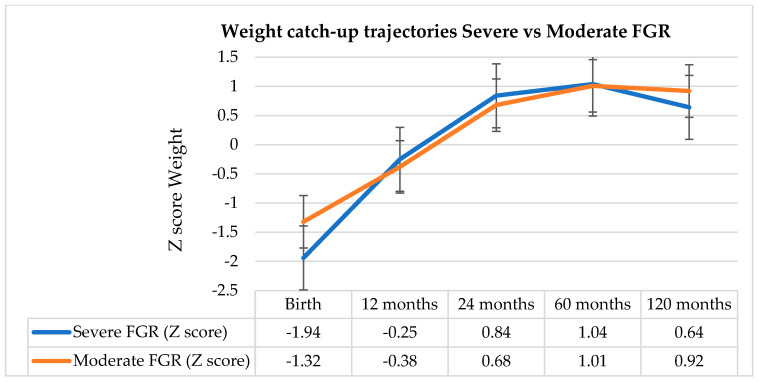
Comparison between mean weight z-scores at ages 1 year, 2 years, 5 years and 10 years, according to FGR severity.

The main effect of time over FGR outcomes was F = 48.341 with *p* < 0.001: this resulted in important differences between the four time points, with Z scores for weight changing significantly from 1 to 10 years. Regarding the interaction between time and severity of FGR, this was not statistically significant (F = 1.395 with *p* = 0.249) and, consequently, we can state that the change in Z scores over time is similar between children with severe and moderate FGR (Figure 5).

**Figure 6:**
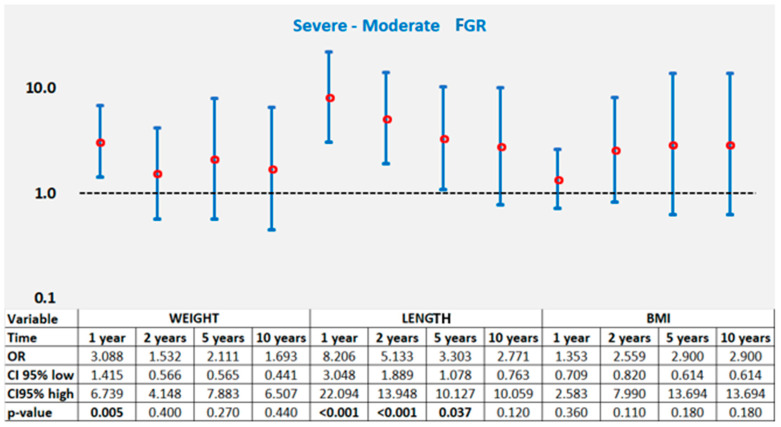
Results of the logistic regression model for catch-up for cumulative weight, length, and BMI for time intervals of 1, 2, 5 and 10 years (univariate analysis).

Across all four points in time, children with severe FGR demonstrated significantly higher proportions in achieving normal weight at 1 year (*p* = 0.005), suggesting a more pronounced early catch-up pattern in their first 12 months of life (Tabel 3, Figure 6).

Severe FGR infants presented shorter birth length (43.57 ± 1.13 cm) compared to those with moderate FGR (46.18 ± 1.14 cm; *p* < 0.001) (Tabel 3). Both groups presented most of their length recovery during the first year (Figure 3). However, the severe FGR group consistently outperformed the moderate group, with markedly higher proportions achieving normal length at 1 and 2 years (both *p* < 0.001) and at 5 years (*p* = 0.037).

Interestingly, BMI trajectories did not differ significantly between the two FGR categories at any age (*p* > 0.05), suggesting that weight-for-length proportionality is restored relatively uniformly across severity groups (Tabel 3, Figure 6).

At 24 months of age, we observed a higher percentage of patients with severe FGR with a Z-score value above 2 (the differences are 12.3% for BMI and 2.8% for weight). In the case of BMI, the percentage difference between patients with severe and moderate FGR of 12.3% was quite close to the significance level of 0.05 used in the study (χ^2^ = 2.963 with *p* = 0.085). At 10 years of age, we observed a reversal of the share held by children with a Z-score above 2—obese—in favor of those with moderate FGR, without, however, these differences (8.3% for BMI and 3.5% for weight) being statistically significant (Table 5).

## 4. Discussion

In this retrospective cohort of term infants affected during the antenatal period by growth restriction, we observed substantial catch-up growth across anthropometric parameters at 4 particular moments during their first decade of life. These results align with previous evidence showing these children’s capacity for rapid catch-up growth following intrauterine constraint, particularly within their first two years of life [58,59,60].

### 4.1. Sex Differences in Growth Trajectories

Consistent with anterior scientific research demonstrating minimal difference in anthropometric parameters among infants born small or growth-restricted, this retrospective cohort of term infants affected during the antenatal period by growth restriction displayed substantial catch-up growth across all anthropometric parameters from birth to 10 years of age.

There were no significant differences in mean weight, length, and BMI among males and females (*p* > 0.05 for all comparisons) at birth. Divergent growth trajectories did, however, appear in the early postnatal period. At one and two years of age, males showed noticeably higher values for BMI and weight, and at one-year, bigger length.

This pattern is consistent with studies documenting the existence of a slightly faster somatic growth in male infants during infancy [59,61]. In a different manner, females achieved significantly higher mean values for weight and BMI at 10 years, with statistically significant elevated weight Z-scores at 60 months. These findings suggest that sexual dimorphism in the growth of FGR-affected children is subtle during infancy but becomes more pronounced during the preschool years.

The accelerated weight gain observed in female sex between 2 and 5 years, maintained through to age 10, aligns with existing evidence that girls experience an earlier adiposity rebound than boys, which may further increase the risk of fat tissue accumulation in later childhood [34,62]. Rambhojan et colleagues [63] demonstrated that girls gain more fat mass approaching puberty, consistent with our findings [63]. Ong et al. [43] reported that early adiposity rebound is associated with an augmented obesity risk in childhood and adolescence, while Ibáñez et al. [45,64], in their two reports, outlined an early fat mass accretion and metabolic alterations in girls born small for their gestational age, who underwent rapid catch-up growth [45,64]. This sex-specific pattern is consistent with our observation that female sex was a significant predictor of increased weight Z-scores at 60 months of life.

Despite these variations in absolute size, we found no sex-related differences in the proportion of children achieving catch-up growth in weight, length, or BMI at any point. Consistent with systematic reviews highlighting that most SGA infants display catch-up growth by age 2 [41], our cohort showed that both males and females attained similar proportions of catch-up in weight and BMI by 24 months, and in linear growth by the age of 5. Accordingly, Karlberg and colleagues [59,60] described the early postnatal period as the critical window for spontaneous recovery regardless of an individual’s sex.

These findings echo earlier reports indicating that male and female infants have a broadly comparable potential to achieve normal weight and height, even if differences in the speed or extent of growth appear later in childhood [44,59,61]. Thus, sexual dimorphism in body measurements appears to reflect typical developmental physiology rather than differences in recovery after intrauterine growth disturbances. Put differently, although sex affects overall size, it does not appear to interfere with the fundamental compensatory growth processes following FGR.

### 4.2. Impact of FGR Severity on Growth Trajectories

Early growth postnatal outcomes were strongly associated with FGR severity. In accordance with the degree of placental insufficiency that characterizes important growth restriction, infants with severe FGR had significantly lower birth weight and length values [65,66]. Despite this initial deficit, infants affected by severe FGR presented more pronounced early recovery growth compared with those with moderate forms of FGR. At 1 and 2 years, they were significantly more likely to recover their age-appropriate weight and length. This pattern mirrors observations from previous studies reporting that once intrauterine constraints are removed, infants that suffered from severe forms of growth impairments often exhibit the most pronounced initial catch-up response [42,52,67].

By the ages of 5 and 10 years, however, we did not notice any significant differences in mean weight, length, or BMI between moderate and severe FGR groups. This alignment of growth curves across these two study groups is strongly supported by the current literature. Both SGA and growth-restricted infants have been shown to undergo important early catch-up growth, especially when they benefit from adequate postnatal nutritional support [41,52]. Similarly, Hendrix et al. [47] and McLaughlin et al. [67] announced that presenting compensatory postnatal growth with the subsequent achievement of normal anthropometric indices by early childhood is a common finding for infants with reduced antenatal growth velocity.

In our study report, BMI trajectories did not differ significantly between the two severity groups at any age. This uniformity in BMI values may be proof of proportional gains in weight and length, as in a symmetrical restoration of growth capacity. However, the equalization of basic anthropometric measures to those of the corresponding age and sex does not necessarily imply normalization of body composition or metabolic risk. Even when weight and height values normalize, prior research shows that children with severe forms of FGR are more likely to achieve more fat mass than lean mass during the growth recovery process [49,57], opening the path to later obesity and insulin resistance [27,62]. Although our BMI data did not differ significantly between severity groups, the higher proportion of severely affected infants with BMI Z-scores >2 at 24 months of age (*p* = 0.085) may signal early disproportionate adiposity gain—a pattern described in both human and animal studies [68,69]. These observations reinforce the hypothesis that BMI alone cannot detect changes in the body composition of children recovering from FGR, as fat mass distribution—particularly visceral adiposity—may vary independently [57,70].

Finally, the severe FGR group demonstrated a better catch-up in height than the moderate group at early time points. This counterintuitive observation is consistent with evidence from discordant twin studies, in which the more growth-restricted co-twin often reaches a more pronounced catch-up in linear growth. These findings support the existence of intrinsic biological mechanisms that promote attainment of an individual’s genetically determined height potential [46].

### 4.3. Catch-Up Growth and Long-Term Health Implications

Despite catch-up growth processes being usually associated with improved neurodevelopment outcomes and reduced morbidity, accelerated postnatal weight gain has been frequently related to adverse long-term metabolic consequences. Several longitudinal studies have shown that rapid early growth following FGR leads to increased adiposity, insulin resistance, and progression to different features of metabolic syndrome during childhood and adolescence [27,46,71]. Furthermore, a recent systematic review and meta-analysis performed by Martín-Calvo et al. [72] established that both SGA status and low birth weight are increasing the risk of obesity in pediatric populations.

Accelerated early weight gain, irrespective of birthweight, is also recognized as a predictor of later overweight and obesity [43,73,74]. Our observation that severe FGR infants exhibit important early catch-up may be related to subsequent long-term metabolic health. Evidence from DOHaD supports the concept that infants exposed to intrauterine nutrient deprivation may undergo adaptive programming, increasing susceptibility to adiposity when postnatal nutrition is abundant [27,58].

These considerations underscore the need for careful growth monitoring, particularly during the first two years of life. Ensuring adequate—but not excessive—nutritional support is essential to encourage healthy growth while mitigating metabolic risk [58]. Lifestyle interventions in school-aged children have shown potential to improve metabolic profiles, highlighting the importance of early identification and follow-up of high-risk children [63].

### 4.4. Linear Growth: Recovery and Determinants

More than two-thirds of children achieved normal length by the age of 1 year, with this value increasing to nearly 90% by 10 years, similar to earlier reports demonstrating that most term-born SGA infants achieve normal stature during childhood [59,60]. However, in our study, children with severe FGR consistently reached normal length proportions earlier than those with moderate FGR, most cases in early infancy, at 1 and 2 years of age.

The determinants of linear catch-up growth are complex. Suboptimal height recovery has been linked to several processes including persistent endocrine dysregulation, chronic inflammation, and postnatal nutritional inadequacy [75]. Some cohorts have not achieved complete catch-up even in adolescence, particularly those under low socioeconomic conditions [76]. The high recovery rates observed in our study suggest that consistent postnatal care and adequate nutritional support may mitigate such risks.

### 4.5. Strengths and Limitations

This study has several notable strengths. The longitudinal design, with periodic standardized measurements of anthropometric parameters from birth through 10 years of age, provides a comprehensive characterization of growth trajectories in children with a history of fetal growth restriction (FGR). The extended follow-up period allowed the evaluation of both early catch-up dynamics and later childhood growth patterns, offering valuable insight into the timing and persistence of sex- and severity-related differences. Furthermore, the use of World Health Organization age- and sex-adjusted z-scores ensured comparability across different ages and minimized bias related to developmental stage. Importantly, the exclusive inclusion of term-born children minimized the confounding influence of prematurity, which is an independent determinant of postnatal growth and body composition. Finally, stratification according to FGR severity enabled a more granular analysis of postnatal recovery in relation to the degree of intrauterine compromise, an aspect that remains underexplored in the existing literature.

However, several limitations must be acknowledged and carefully considered when interpreting our findings. First, the study did not include a non-FGR control group of children born appropriate for gestational age, which limits the ability to fully contextualize the observed growth trajectories relative to typical postnatal development. Although standardized reference values were used, direct comparison with a contemporaneous control group would have strengthened causal inference regarding the specific impact of FGR on postnatal growth patterns.

Second, body composition data—including fat mass, lean mass, and regional or visceral adiposity—were not available. While BMI and weight-for-age are widely used and clinically practical indicators, they do not differentiate between qualitative components of growth. This limitation is particularly relevant in children recovering from FGR, as prior evidence suggests that catch-up growth in this population may preferentially involve fat mass accretion rather than proportional gains in lean tissue [34,35]. Consequently, the normalization of BMI observed in our cohort does not necessarily imply normalization of body composition or metabolic risk, and subtle differences in adiposity distribution may have remained undetected.

Third, information on several important confounding variables was unavailable. Detailed data on nutritional intake during infancy and childhood—including breastfeeding duration, formula use, complementary feeding practices, and overall caloric intake—were not captured. Postnatal nutrition plays a central role in determining the timing and magnitude of catch-up growth and may substantially modify long-term metabolic outcomes [72]. Similarly, physical activity levels were not assessed, limiting our ability to evaluate lifestyle-related modifiers of growth and body composition, particularly at later childhood time points. Physical activity is increasingly recognized as a key modifier of childhood body composition and cardiometabolic risk, particularly in school-aged children. Differences in habitual activity patterns could influence BMI trajectories and fat distribution independently of early life growth restriction [73,74].

Parental anthropometry was also not available for inclusion in the analysis. Parental height and weight influence offspring growth trajectories through genetic and shared environmental pathways and are especially relevant when interpreting linear catch-up growth and final height attainment [10]. Without these data, it is not possible to fully distinguish genetically determined growth potential from compensatory postnatal growth following intrauterine restriction. In addition, pubertal timing was not captured in the available dataset. Puberty represents a critical period of accelerated growth and profound hormonal changes that can significantly affect height, weight, and body composition [35]. Variability in the onset and tempo of puberty, particularly between sexes, may influence anthropometric measurements at 10 years of age and contribute to the observed sex-related differences in weight and BMI. The absence of pubertal staging limits our ability to fully interpret late childhood growth patterns and underscores the need for cautious interpretation of anthropometric differences observed near the peripubertal period.

Finally, information regarding FGR etiology and maternal comorbidities was limited. FGR is a heterogeneous condition arising from diverse maternal, placental, and fetal factors, and different etiological pathways may confer distinct postnatal growth and metabolic risks [10]. The inability to stratify outcomes according to underlying FGR mechanisms may have obscured important biological variability within the cohort.

Despite these limitations, the primary objective of the study—to characterize longitudinal anthropometric growth trajectories and catch-up growth patterns in term-born children with FGR according to sex and severity rather than to directly assess metabolic outcomes or body composition—remains valid. The absence of body composition measurements and certain confounding variables represents a limitation of the present study, but these constraints do not undermine its central contribution. The findings should therefore be interpreted within the context of these acknowledged constraints. Future prospective studies incorporating detailed nutritional assessments, objective measures of physical activity, parental anthropometry, pubertal staging, etiological classification of FGR, and direct body composition measurements will be essential to further elucidate the long-term health implications of growth recovery following FGR.

## 5. Conclusions

This longitudinal cohort study offers proof that FGR severity degree represents a key determinant of early postnatal growth, with infants affected by severe FGR presenting accelerated catch-up in weight and length during the first two years of life. By mid-childhood, growth trajectories converged across severity groups, and BMI recovery was uniformly achieved regardless of initial intrauterine constraint. Sex did not influence the likelihood or timing of catch-up growth, although expected physiological differences in absolute anthropometric values emerged over time.

These findings reinforce the substantial capacity for growth recovery following FGR while highlighting the critical importance of monitoring early postnatal growth to ensure adequate—but not excessive—catch-up. Given the well-established associations between rapid weight gain and later metabolic risk, long-term surveillance into adolescence and adult age remain essential. Continued follow-up of this cohort will help clarify whether early normalization of anthropometrics translates into sustained metabolic health.

## Figures and Tables

**Table 1:** Baseline characteristics of the study group.

Variable	Total	Male	Female	*p* Value	Moderate FGR	Severe FGR	*p* Value
Cases	170	68 (40%)	102 (60%)		110 (64.61%)	60 (35.29%)	
Birth weight (g)	2.51 ± 0.19	2.52 ± 0.17	2.51 ± 0.19	0.67	2.61 ± 0.10	2.32 ± 0.16	**<0.001**
GA at birth (weeks)	38 ± 0.51	38 ± 0.54	38 ± 1.78	1.000	38 ± 0.96	38 ± 0	1.0
Natural vaginal delivery	2 (1.2%)	0	2 (1.96%)		0	2 (3.33%)	
Cesarean delivery	168 (98.8%)	68 (100%)	100 (98.94%)		110 (100%)	58 (96.67%)	
Maternal age at delivery (years)	28.44 ± 6.79	27.98 ± 5.57	28.75 ± 7.50	0.381631	28.52 ± 6.80	28.3 ± 6.81	0.818353197
Maternal education < 4 classes	29 (17.05%)	18 (26.47%)	11 (10.78%)		23 (20.9%)	6 (10%)	
Maternal education > 12 classes	14 (8.23%)	5 (7.35%)	9 (8.82%)		8 (7.27%)	6 (10%)	
Birth length (cm)	45.26 ± 1.69	45.43 ± 1.65	45.15 ± 1.71	0.29	46.18 ± 1.14	43.57 ± 1.13	**<0.001**
Male sex	68 (40%)	68 (100%)	/		45 (40.9%)	23 (38.33%)	
Breastfeed within first 6 months of life	54 (31.77%)	21 (30.88%)	33 (32.35%)		32 (29.1%)	22 (36.66%)	
Formula fed within first 6 months of life	80 (47.05%)	34 (50%)	46 (45.1%)		53 (48.18%)	27 (45%)	
Mixed alimentation within first 6 months of life	36 (21.18%)	13 (19.12%)	23 (22.55%)		25 (22.72%)	11 (18.34%)	
Rural area	126 (74.11%)	53 (77.94%)	73 (71.56%)		79 (71.81%)	47 (78.33%)	
Urban area	44 (25.89%)	15 (22.06%)	29 (28.44%)		31 (28.19%)	13 (21.67%)	

GA: Gestational age. <4 classes refer to primary cycle of education. >12 classes refer to post-secondary/higher education. Available data were displayed either as number and percent or as mean value and standard deviation. *p* value < 0.05, marked in bold font, was considered as a significant difference between two groups.

**Table 2:** Description of variables weight, length, and BMI (mean and standard deviation) at 1, 2, 5 and 10 years.

Variable	Total	Male	Female	*p* ^a^	Moderate FGR	Severe FGR	*p* ^b^
Cases	170	68	102		110	60	
Weight (kg)							
Initial	2.51 ± 0.19	2.52 ± 0.17	2.51 ± 0.19	0.67	2.61 ± 0.10	2.32 ± 0.16	**<0.001**
1 year	8.83 ± 0.99	9.22 ± 1.09	8.57 ± 0.83	**<0.001**	8.79 ± 1.01	8.90 ± 0.98	0.49
2 years	12.69 ± 1.62	13.04 ± 1.55	12.45 ± 1.63	**0.02**	12.63 ± 1.56	12.79 ± 1.74	0.52
5 years	20.11 ± 4.36	19.63 ± 4.23	20.42 ± 4.44	0.25	20.09 ± 4.32	20.13 ± 4.47	0.96
10 years	36.42 ± 11.19	34.33 ± 8.34	37.81 ± 12.59	**0.03**	36.93 ± 11.46	35.47 ± 10.69	0.42
Length (cm)							
Initial	45.26 ± 1.69	45.43 ± 1.65	45.15 ± 1.71	0.29	46.18 ± 1.14	43.57 ± 1.13	**<0.001**
1 year	73.22 ± 2.53	73.88 ± 2.06	72.77 ± 2.71	**0.003**	73.32 ± 2.53	73.02 ± 2.53	0.45
2 years	84.30 ± 3.27	84.59 ± 2.51	84.11 ± 3.69	0.31	84.44 ± 3.11	84.05 ± 3.56	0.46
5 years	109.22 ± 7.24	108.12 ± 5.59	109.96 ± 8.10	0.08	109.31 ± 7.18	109.07 ± 7.42	0.84
10 years	142.79 ± 10.37	143.79 ± 7.89	142.12 ± 11.73	0.27	143.34 ± 9.24	141.78 ± 12.20	0.39
BMI (kg/m^2^)							
Initial	12.24 ± 0.41	12.19 ± 0.33	12.27 ± 0.45	0.21	12.25 ± 0.37	12.23 ± 0.47	0.83
1 year	16.44 ± 1.33	16.86 ± 1.54	16.16 ± 1.08	**<0.001**	16.32 ± 1.37	16.66 ± 1.23	0.11
2 years	17.81 ± 1.68	18.20 ± 1.87	17.55 ± 1.49	**0.01**	17.67 ± 1.67	18.06 ± 1.67	0.15
5 years	16.76 ± 2.68	16.78 ± 3.33	16.75 ± 2.16	0.948	16.74 ± 2.76	16.81 ± 2.56	0.86
10 years	17.63 ± 3.84	16.55 ± 3.56	18.35 ± 3.87	**0.002**	17.73 ± 3.95	17.45 ± 3.67	0.65

In bold, significant differences (*p* value < 0.05). ^a,b^ Comparison between the two study groups (^a^ male vs. female or ^b^ moderate vs. severe FGR).

**Table 3:** Cumulative catch-up for weight, length, and BMI, with repeated measures assessment at 1, 2, 5 and 10 years.

Variable	Total	Male	Female	*p* ^a^	Moderate FGR	Severe FGR	*p* ^b^
Cases	170	68	102		110	60	
Weight (kg)							
1 year	118 (69.41%)	50 (73.53%)	68 (66.67%)	0.34	68 (61.82%)	50 (83.33%)	**0.005**
2 years	148 (87.06%)	60 (88.24%)	88 (86.27%)	0.71	94 (85.45%)	54 (90.00%)	0.40
5 years	156 (91.76%)	63 (92.65%)	93 (91.18%)	0.73	99 (90.00%)	57 (95.00%)	0.27
10 years	158 (92.94%)	64 (94.12%)	94 (92.16%)	0.63	101 (91.82%)	57 (95.00%)	0.44
Length (cm)							
1 year	118 (69.41%)	48 (70.59%)	70 (68.63%)	0.79	63 (57.27%)	55 (91.67%)	**<0.001**
2 years	130 (76.47%)	56 (82.35%)	74 (72.55%)	0.14	75 (68.18%)	55 (91.67%)	**<0.001**
5 years	145 (85.29%)	60 (88.24%)	85 (83.33%)	0.38	89 (80.91%)	56 (93.33%)	**0.037**
10 years	153 (90.00%)	64 (94.12%)	89 (87.25%)	0.15	96 (87.27%)	57 (95.00%)	0.12
BMI (kg/m^2^)							
1 year	63 (37.06%)	27 (39.71%)	36 (35.29%)	0.56	38 (34.55%)	25 (41.67%)	0.36
2 years	149 (87.65%)	60 (88.24%)	89 (87.25%)	0.85	93 (84.55%)	56 (93.33%)	0.11
5 years	158 (92.94%)	62 (91.18%)	96 (94.12%)	0.47	100 (90.91%)	58 (96.67%)	0.18
10 years	158 (92.94%)	62 (91.18%)	96 (94.12%)	0.47	100 (90.91%)	58 (96.67%)	0.18

In bold, significant differences (*p* value < 0.05). A gain greater than 0.67 in weight, length, and BMI z-scores in the growth curves in any of these periods was considered significant catch-up. ^a,b^ Comparison between the two study groups (^a^ male vs. female or ^b^ moderate vs. severe FGR).

**Table 4:** Gender comparisons of the percentages of the 170 patients with Z-scores above 2 (for weight, height and BMI).

	Male	Female	$\chi^{2}\;Test$/Fisher *	*p* Value
**Weight**				
Z 120 months	16.2%	23.5%	1.349	0.245
**Z 60 months**	**13.2%**	**33.3%**	8.722	**0.003**
Z 24 months	13.2%	18.6%	0.862	0.353
**Height**				
Z 120 months	14.7%	18.6%	0.443	0.505
Z 60 months	2.9%	7.8%	-	0.319
Z 24 months	-	-	-	-
**BMI**				
Z 120 months	13.2%	19.6%	1.171	0.279
**Z 60 months**	**13.2%**	**25.5%**	3.748	**0.053**
Z 24 months	33.8%	22.5%	2.628	0.105

* Fisher’s test was used due to the presence of expected values below 5. In bold, significant differences (*p* value < 0.05).

**Table 5:** Comparisons between the 2 types of FGR (severe and moderate) of the percentages of the 170 patients with Z-scores above 2 (for weight, height and BMI).

	Severe FGR	Moderate FGR	$\chi^{2}\;Test$/Fisher *	*p* Value
**Weight**				
Z 120 months	18.3%	21.8%	0.288	0.591
Z 60 months	28.3%	23.6%	0.453	0.501
Z 24 months	18.3%	15.5%	0.234	0.629
**Height**				
Z 120 months	16.7%	17.3%	0.010	0.920
Z 60 months	8.3%	4.5%	-	0.326
Z 24 months	-	-	-	-
**BMI**				
Z 120 months	11.7%	20.0%	1.906	0.167
Z 60 months	18.3%	21.8%	0.288	0.591
**Z 24 months**	**35.0%**	**22.7%**	2.963	**0.085**

* Fisher’s test was used due to the presence of expected values below 5. In bold, significant differences (*p* value < 0.05).

## Data Availability

The data presented in this study are available on request from the corresponding author due to privacy.

## Abbreviations

The following abbreviations are used in this manuscript:

FGR	Fetal growth restriction
IUGR	Intrauterine growth restriction
WHO	World Health Organization
NAFLD	Non-alcoholic fatty liver disease
DOHaD	Developmental Origins of Health and Disease
SGA	Small for gestational age
BMI	Body mass index
GA	Gestational age
